# Solid-state calculations for iterative refinement in quantum crystallography using the multipole model

**DOI:** 10.1107/S2052252525002040

**Published:** 2025-04-04

**Authors:** Michael Patzer, Christian W. Lehmann

**Affiliations:** a Chemische Kristallographie und Elektronenmikroskopie, Max-Planck-Institut für Kohlenforschung, Kaiser-Wilhelm-Platz 1, Mülheim an der Ruhr, 45470North Rhine-Westphalia, Germany; ESRF, France

**Keywords:** quantum crystallography, multipole model, *ReCrystal*, *CRYSTAL17*, solid-state calculations, transferable atom form factors, charge density analysis

## Abstract

A quantum crystallographic refinement methodology has been developed using theoretical multipole parameters generated directly from solid-state calculations using the *CRYSTAL17* program. This refinement method is comparable to other transferable form factor approaches, such as the Invariom model, but in contrast to the Hirshfeld atom refinement, it uses theoretical multipole parameters to describe the electron density from a solid-state calculation performed with *CRYSTAL17* in an iterative refinement procedure. For this purpose, a Python3 code named *ReCrystal* has been developed. To start *ReCrystal*, a CIF, a Gaussian basis set, a DFT functional and the number of CPUs must be defined. The Pack–Monkhorst and Gilat shrinking factors, which define a lattice in the first Brillouin zone, must also be specified. After *k*-point sampling, *CRYSTAL17* calculates structure factors directly from the static electron density. Multipole parameters are generated from these structure factors using the *XD* program and are fixed during least-squares refinement. The refinement of the xylitol molecular crystal has shown that the hydrogen atom positions can be determined with reasonable agreement to those obtained in the neutron diffraction experiment. This indicates that the periodic boundary condition in *ReCrystal* is an improvement over gas phase refinement with HAR. The multipole parameters obtained from *ReCrystal* can be used for further charge density studies especially if weak interactions are the focus. In this work, we demonstrate the performance of *ReCrystal* on molecular crystals of the small molecules d/l-serine and xylitol with weak hydrogen-bonding motifs using multipole refinement. The advantage of this approach is that multipole parameters can be obtained from high-resolution calculated diffraction data, no database is required, and errors due to the model and errors resulting from the experiment are clearly separated.

## Abstract 2

This work presents a new iterative refinement method, comparable to Hirshfeld atom refinement, using the Hansen–Coppens multipole model charge density description to obtain accurate atomic coordinates and atomic displacements based on *CRYSTAL17* periodic boundary calculations. The refinement, performed using the Python code *ReCrystal*, allows the user to explore the full periodic charge density in the crystalline solid state for charge density analysis of weak interactions.

## 1. Introduction

Quantum crystallographic (QCr) refinement techniques for single-crystal X-ray diffraction have undergone a revolutionary development in the last decades and have made a fundamental contribution to the understanding of the diffraction experiment in single-crystal structure analysis (Grabowsky *et al.*, 2017 ▸; Genoni *et al.*, 2018 ▸). These methods have led to a remarkable improvement of the refinement results due to the use of aspherical atomic form factors (Dittrich *et al.*, 2005 ▸; Dittrich *et al.*, 2006 ▸; Dominiak *et al.*, 2007 ▸; Jayatilaka & Dittrich, 2008 ▸; Capelli *et al.*, 2014 ▸). Single-crystal structure analysis applies a model of the charge density distribution in the unit cell to obtain information about the phases of the intensities in the diffraction pattern. This model is related to the diffraction intensities via a Fourier transform. The purpose of the refinement is to find a model which best describes the intensities of the reflections. The core idea of the advanced refinement methods is to calculate atomic scattering factors from charge densities calculated by quantum mechanics. The basic assumption is that the charge density derived from theoretical calculations is in good agreement with the real density given in the crystal. A significant advantage of QCr methods is that they do not require high-resolution data (*d* < 0.5 Å) like in a conventional charge density analysis to obtain very precise information about the positions and displacements of the atoms in the crystal, especially of the light atoms. Nevertheless, it should be emphasized that high-resolution diffraction data are essential in order to obtain an accurate crystal structure using single-crystal X-ray diffraction.

For QCr refinement, a partitioning scheme is required, and two leading methods are discussed in this paper. In Hirshfeld atom refinement (HAR), the Hirshfeld partitioning scheme defines the electron density of an atom (Jayatilaka & Dittrich, 2008 ▸; Capelli *et al.*, 2014 ▸). This method, which first appeared in the program *TONTO* (Jayatilaka & Grimwood, 2003 ▸) can nowadays be used in other structure refinement programs such as *NoSpherA2*/*OLEX2* (Kleemiss *et al.*, 2021 ▸), *DiSCaMb* (Chodkiewicz *et al.*, 2018 ▸) or *lamaGOET* (Malaspina *et al.*, 2021 ▸). Coordinates and anisotropic displacement parameters of hydrogen atoms can be determined for a number of examples with an accuracy that is otherwise only observed in neutron diffraction experiments (Ruth *et al.*, 2022 ▸; Chodkiewicz *et al.*, 2020 ▸; Woińska *et al.*, 2021 ▸). However, HAR is not yet a reliable substitute for neutron measurements in general, as it has been shown that it is still not possible to find suitable models for all systems and modifications are required (Chodkiewicz & Woźniak, 2025 ▸). For this reason, intensive research is continuing on the method in order to optimize this approach. Work has been done in electron correlation handling. In principle, any theoretical method that differs in the treatment of electron correlation can be used to calculate a charge density (*e.g.* Hartree–Fock, DFT, MP2, CCSD). However, previous studies have already shown that the model charge density obtained from a DFT calculation is a good approximation for QCr refinement (Wieduwilt *et al.*, 2020 ▸). DFT in HAR has been shown to differ significantly when the Hartree–Fock exchange is varied (Landeros-Rivera *et al.*, 2023 ▸). Another sophistication is to include the normal modes from a solid-state calculation in the refinement. In this procedure the frequencies of the modes are refined and a precise determination of the anisotropic displacement parameters is possible (Hoser & Madsen, 2016 ▸, Butkiewicz *et al.*, 2025 ▸). The influence of the partitioning scheme on the refinement result has been investigated and the advantages of alternative approaches have been analysed (Chodkiewicz *et al.*, 2020 ▸; Chodkiewicz & Woźniak, 2025 ▸). Until very recently, most HAR investigations use gas phase calculations with programs such as *ORCA* (Neese, 2022 ▸), *Gaussian* (Frisch *et al.*, 2016 ▸), *pySCF* (Sun *et al.*, 2020 ▸) or *TONTO* (Jayatilaka & Grimwood, 2003 ▸) being employed to calculate the aspherical atomic form factors. The importance of intermolecular interactions for the understanding of a solid in its physical and chemical properties is widespread in crystallography (Deringer *et al.*, 2017 ▸) and is particularly emphasized in this work. In HAR applications, cluster charges and dipoles can be used to describe the effects of the environment on the charge density of a molecule (Woińska *et al.*, 2014 ▸). An alternative to this HAR approach, which has so far been used scarcely, is the calculation under periodic boundary conditions (Ruth *et al.*, 2022 ▸; Wall, 2016 ▸). Recently, Dietmar Stalke’s group presented a plane wave based method for HAR (PAW-HAR) and could show the benefit of periodic boundaries (Ruth *et al.*, 2022 ▸).

In this work, we report an iterative refinement procedure which uses periodic boundary theoretical calculations with *CRYSTAL17* (Dovesi *et al.*, 2018 ▸) to create a multipole description of the electron density for use in iterative least-squares refinement of X-ray diffraction data. We show that when using this method for d/l-serine and xylitol the hydrogen atoms in the hydrogen bonds are well described. In the case of xylitol, an improvement in the hydrogen atom positions compared with the gas phase HAR is observed using neutron diffraction results as a reference. This paper presents a method to independently compare the charge density from a solid-state calculation with the experimental charge density, thus allowing accurate comparisons between theory and experiment. For this purpose, the Hansen–Coppens multipole model is used, which has shown in numerous cases an inferior performance compared with the HAR approach in determining hydrogen atom positions (Chodkiewicz *et al.*, 2024 ▸; Woińska *et al.*, 2014 ▸). However, there is a weakness in the HAR with regards to the derivation of atomic displacement parameters compared with the multipole models (Köhler *et al.*, 2019 ▸).

The Hansen–Coppens multipole model, developed in the 1970s for crystallographic refinement, describes the aspherical charge density of an atom via spherical harmonics and radially adjustable functions (Hansen & Coppens, 1978 ▸), leading to a description of the charge density via a set of multipole model parameters (MMPs). These MMPs can be determined directly from the diffraction experiment or from theoretically calculated structure factors. When fitted experimentally, this is referred to as a charge density study. Models have been developed using transferable MMPs derived from a theoretical calculation, the transferable aspherical atom model (TAAM), such as the Invariom model (Jha *et al.*, 2020 ▸; Dittrich *et al.*, 2004 ▸). However, there are certain limitations with the current multipole-based methods due to need for a reference to a database. This disadvantage can be circumvented by directly calculating the structure factors from the wavefunction. The *CRYSTAL* program offers the possibility of calculating dynamic and static structure factors from the wavefunction (Erba *et al.*, 2013 ▸). These can then be converted into theoretical MMPs (tMMPs). Such parameters can subsequently be used to refine the crystal structure, as in the TAAM or Invariom approach. By employing *CRYSTAL* to calculate the model it is possible to take into account the periodic effects on the charge density. Most recently, an application of TAAM was also developed to use multipole parameters generated directly from a solid-state calculation (Olech *et al.*, 2024 ▸). However, in this case a geometry from the literature is used and therefore information about the molecular structure is required.

## 2. Methodology

In order to coordinate all steps from the generation of the aspherical atomic form factors to the refinement and convergence test, a Python3 script was written, which has been given the name *ReCrystal* (refinement with *CRYSTAL17*). *ReCrystal* (Patzer, 2023 ▸) refers to a multipole model-based refinement in which optimized coordinates, harmonic oscillations and multipole parameters are generated with the help of periodic wavefunctions from the *CRYSTAL17* program (Dovesi *et al.*, 2018 ▸). At this point, note that *ReCrystal* does not claim to be an error-free refinement software, but rather serves as a tool for testing the approach. The *ReCrystal* program used for the project can be found on GitHub (https://github.com/MichaelPatzer/ReCrystal).

### 2.1. Generation of aspherical atom form factors

**Figure 1:**
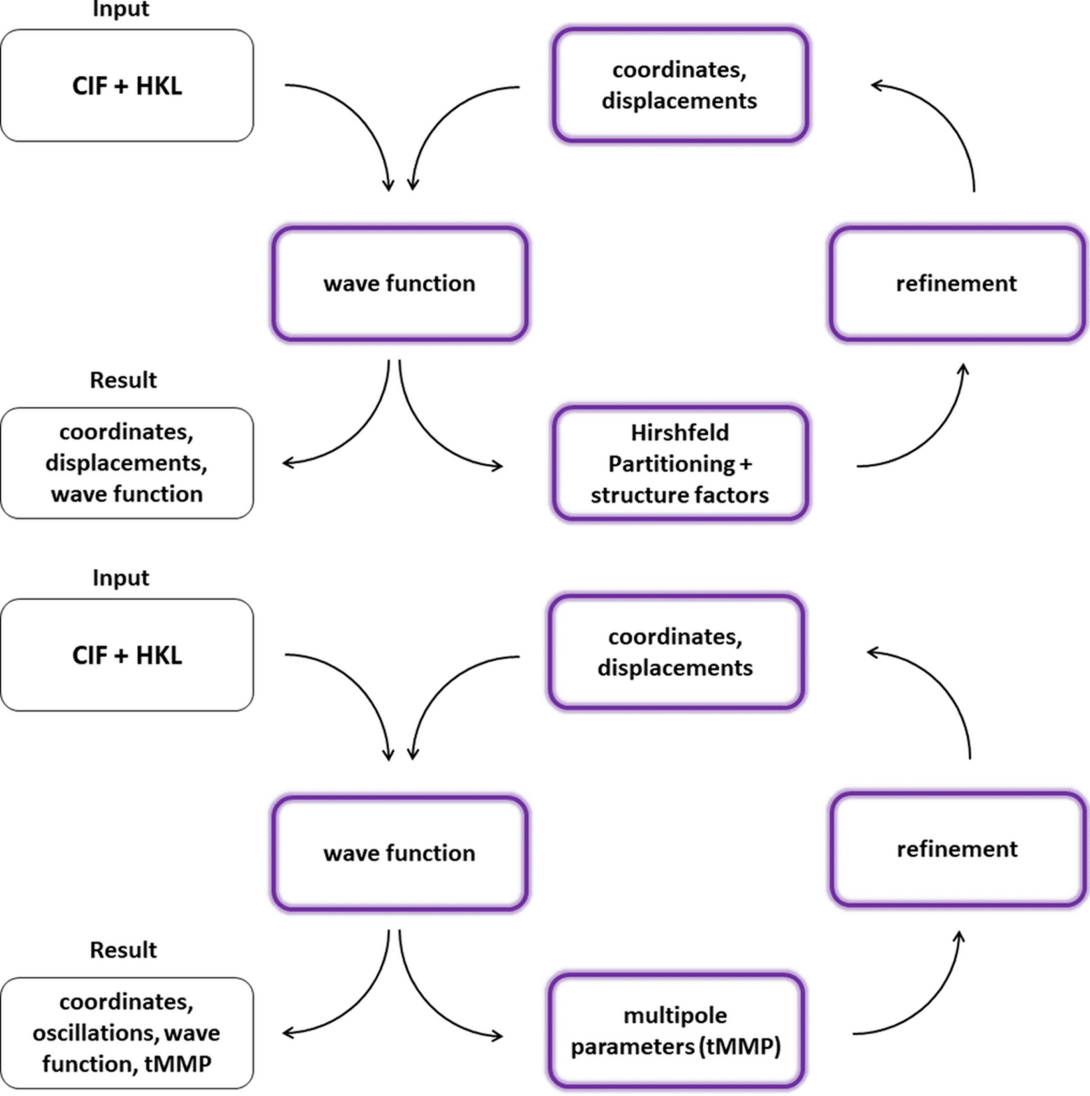
Comparison of the HAR (top) and *ReCrystal* (bottom) procedures. The methods differ mainly in the treatment of the aspherical atomic charge density. The former method uses Hirshfeld atoms. The latter method uses multipole parameters from the Hansen–Coppens multipole model. (1) Generation of a (periodic) wavefunction; (2) generation of aspherical atomic form factors; (3) refinement of *x*, *y*, *z* and ADPs with fixed theoretical aspherical atomic form factors; and (4) convergence test: negative: calculation of new periodic wavefunction using the refined (*x*, *y*, *z*) values, positive: end of refinement.

In the *ReCrystal* approach the atomic charge density in the crystal is described using the Hansen–Coppens multipole model [equation (1)]. Theoretical multipole parameters can be derived from theoretical static structure factors calculated directly by the *CRYSTAL17* program. To evaluate the influence of resolution on the refinement indicators, *ReCrystal* was run at different resolutions (POB-TZVP/PBE). The main conclusion is that there is no significant change in the final *R* value and the resolution of the theoretical intensities in the range 0.9–0.3 Å (see next section). This observation can be explained by the fact that the multipole parameters in the main describe the valence electron density. The value of 0.8 Å has proven to be the most reliable resolution, so that an efficient convergence is found in the *ReCrystal* refinement resulting a low *R*1 value. Due to the fact that the results may differ slightly, we have decided to always specify the resolution used to calculate the tMMP in the *ReCrystal* refinement. Nevertheless, in order to obtain the tMMP with the highest possible accuracy and precision (low standard uncertainties in the refinement), the ratio of theoretical reflections to parameters should be as high as possible.
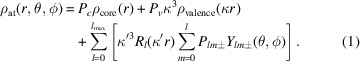
The remaining procedure is almost the same as for HAR (see Fig. 1 ▸). The user of the *ReCrystal* software must select the basis set (*e.g.* POB-TZVP, def2-SVP, def2-TZVP) and a DFT functional before starting the refinement. In addition, the accuracy of the grid in the first Brillouin zone must be defined for the calculation of the *k*-points according to Pack–Monkhorst and Gilat. The resolution of the synthetic dataset used to obtain the tMMP is automatically set to the desired value. The multipole refinement is carried out with the program *XD2006* (Volkov *et al.*, 2006 ▸; https://xd.chem.buffalo.edu/). For the treatment of the radial components in the multipole model, the unpublished VM database contained in *XD2006* is used. For this database, the individual exponents are derived from the density fit for each given orbital. The generation of the tMMP is done step by step to facilitate convergence. The weighting scheme, which is also implemented in *SHELXL*, is used for the refinement on *F*^2^ (see the supporting information, *xd.mas*). In the first part, the multipole occupations of all atoms are refined up to the octupole level. In the second step, the kappa parameters are refined individually for each atom and the multipole occupations are fixed. Finally, the kappa parameters and all multipole parameters are refined together. The multipoles of the hydrogen atoms are fixed in the third step to reduce correlation effects. The scale factor is refined in all steps of the least-squares procedure.

### 2.2. Crystal structure refinement

In this step, the objective is to refine the three positional coordinates *x*, *y* and *z* and the six anisotropic displacement parameters of the atoms in the asymmetric unit. The theoretical multipole parameters are included in the refinement with fixed values and describe the static charge density in the crystal. The experimental diffraction intensities are used as a reference in the refinement. The weighting scheme, which is also implemented in *SHELXL*, is used for the refinement on *F*^2^ (see the supporting information, *xd.mas*). The dispersion correction is switched on again in this step and the tabulated element-specific values implemented in *XD2006* are used for *f*′ and *f*′′ (default Mo *K*α radiation). The entire refinement is again executed in three steps. In the first step, the coordinates and harmonically anisotropic displacements of all atoms heavier than hydrogen are refined. The coordinates and displacement parameters of the hydrogen atoms are not refined in this step. In the second step, the coordinates of the hydrogen atoms are added to the refinement and the displacement parameters are included as isotropic. Finally, all coordinates are freely refined and the displacements of all atoms are treated anisotropically. This three-step procedure is intended to counteract convergence problems due to high correlations. The scale factor is refined in all least-squares procedures. The refined coordinates are checked for convergence in the next step.

**Figure 2:**
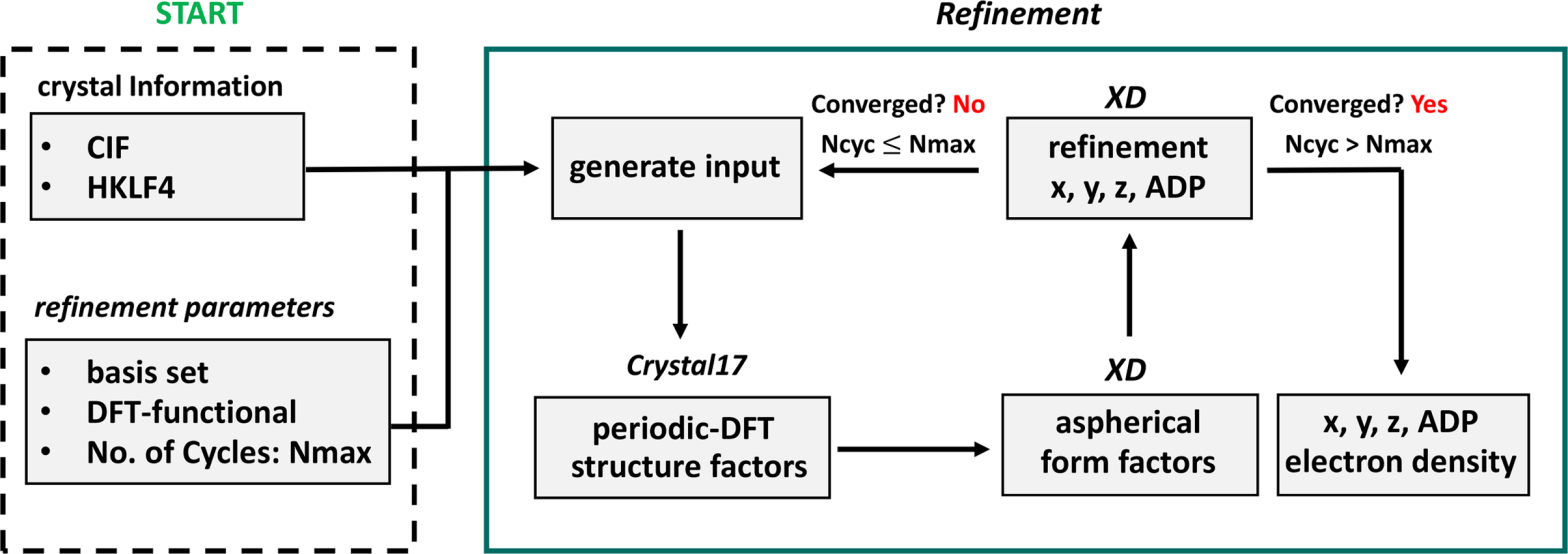
Detailed representation of the sequence of a *ReCrystal* refinement. Initial parameters must be defined once; (1) generation of a periodic wavefunction (2) generation of static structure factors and tMMPs; (3) refinement of *x*, *y*, *z* and ADPs with fixed theoretical MMPs; (4) convergence test: negative, then a new periodic wavefunction is calculated using the refined *x*, *y*, *z* values; positive: end of *ReCrystal* refinement; *N*_cyc_: cycle number; *N*_max_: maximum of *N*_cyc_ defined by user.

For checking convergence, the current version of *ReCrystal* uses the parameter φ_diff_ [equation (2)]. This value checks the geometrically averaged change of coordinates in the entire asymmetric unit after a refinement step. If the value of φ_diff_ is less than 1 × 10^−4^ Å, the refinement is considered to be finished. This means that the coordinates have changed by less than 0.1 mÅ on average within the refinement cycle. Convergence is controlled by the value of φ_diff_ in the *ReCrystal* program from the second cycle onwards.
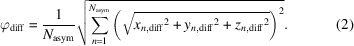
In addition, the atom-specific mean-squared-displacement amplitude for the coordinates and anisotropic displacement parameters are calculated and documented in the output files. If the convergence criterion is not fulfilled, a new periodic wavefunction is computed using the new refined coordinates. The refinement process is repeated (Fig. 2 ▸).

## 3. d/l-serine: basis set and DFT-functional dependence

**Figure 3:**
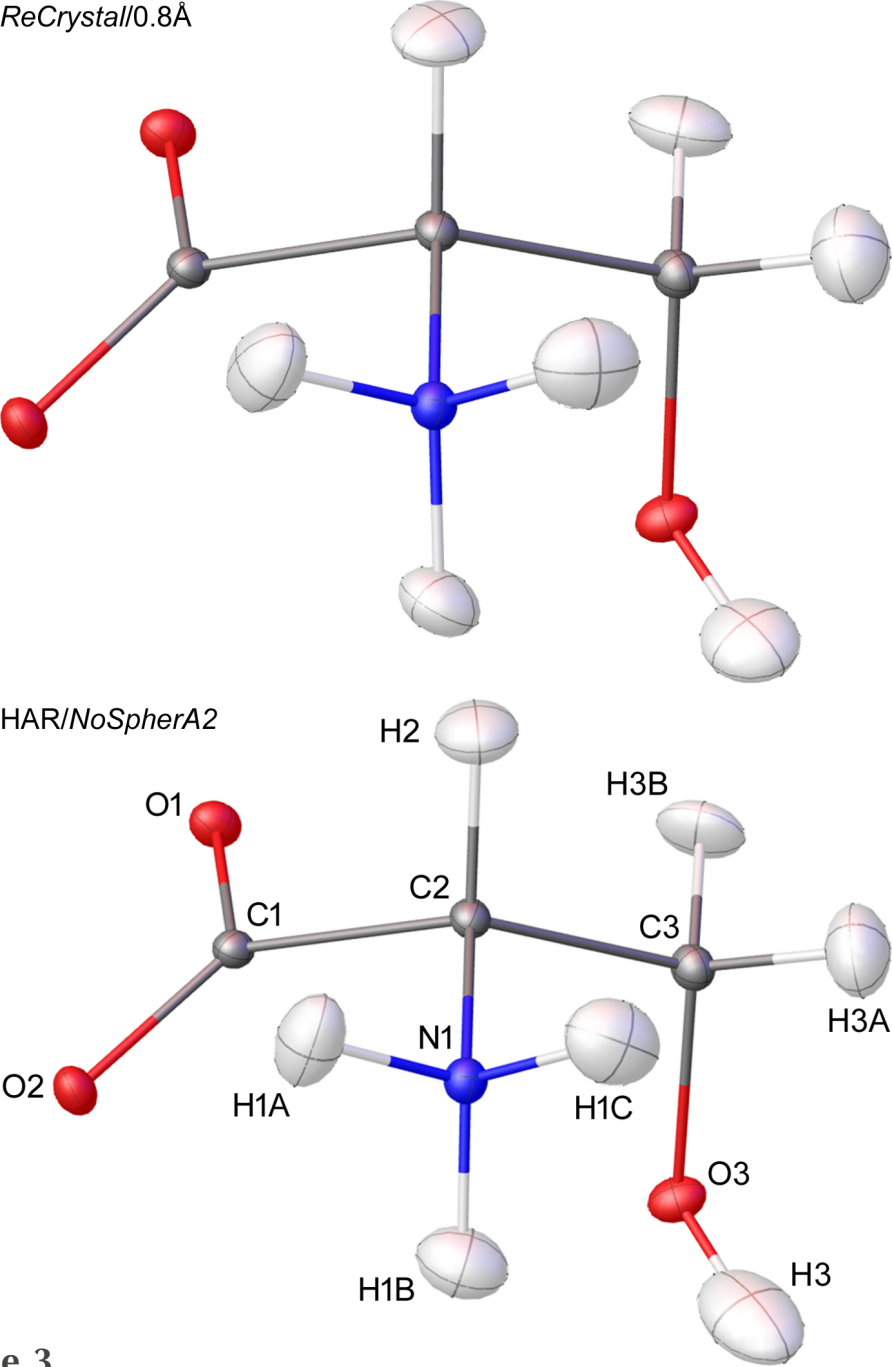
Atomic displacement ellipsoid plot for refinement with *ReCrystal*/0.8 Å (top), NoSphereA2 [*ORCA* (version 5.0.3); bottom] with the basis set def2-TZVP and functional PBE; ellipsoid representation at the 50% probability level (hydrogen – white, carbon – grey, nitro­gen – blue, oxygen – red).

A key issue to be addressed is the influence of the basis set and the DFT functional on the *ReCrystal* refinement result. For this purpose, the X-ray diffraction 100 K dataset of d/l-serine by Peter Luger’s group (Dittrich *et al.*, 2005 ▸) was refined with various basis sets and functionals (Table 1 ▸, CCDC No. 273585). Note that in some cases there were convergence problems on the part of *CRYSTAL17* or *XD*, so that not all combinations in Table 1 ▸ can be assigned an *R* value. *XD* was unable to generate the theoretical multipole parameters from a charge density calculated with a small basis set. This includes the MINIX and STO 3G basis sets. It is assumed that especially the high-angle reflections, which can be assigned to the charge density close to the nucleus, deviate strongly from the expected value due to the small size of the Gaussian basis set. As a result, due to the inadequate description of the near-nuclear charge density, the convergence in the program *XD* is not fulfilled when generating the multipole parameters in *ReCrystal*. When combining the basis set def2-TZVP with the hybrid functionals, such as B3LYP, wb97 or M062X, no convergence was achieved in *CRYSTAL17-SCF*. The combination of the PBE and BLYP functional and the def2-TZVP basis set provides the best *R*1 value of 1.87%. Refining the structure with tMMP generated with intensities up to a resolution of 0.8 Å resulted in an *R*1 of 1.82%. After testing resolutions to generate tMMP between 0.3 and 0.9 Å, the best *R*1 was found at a resolution of 0.8 Å. In view of this variation, we suggest that the resolution to which the theoretical calculations were undertaken in the *ReCrystal* refinement should be given [*ReCrystal*/(number) Å]. Thus, for example, (ReCrystal/0.8 Å). Additionally, it is recommended to test a *ReCrystal* refinement with the Ahlrich basis set def2-TZVP, but keep in mind that the convergence in *CRYSTAL17* might not be fulfilled due to the linear dependencies. The crystal structure was refined with both *ReCrystal*/0.8 Å and *NoSpherA2*/HAR and no significant differences can be recognized between the atomic displacement ellipsoid plots, except perhaps for the directions of the principal axes of the atomic displacement ellipsoids for the hydrogen atoms (Fig. 3 ▸). In the HAR, the ellipsoid of the hydrogen atom H3 is elongated in the direction of the hydrogen bond compared with *ReCrystal*/0.8 Å. A comparison with neutron diffraction results provides additional insight into the advantage of the method (see Section 5 on xylitol). The 0.8 Å value refers exclusively to the resolution of the synthetic dataset of diffraction intensities used to determine the theoretical multipole parameters. The experimental dataset is not truncated and its full resolution is used to determine the displacement parameters and atomic coordinates.

**Figure 4:**
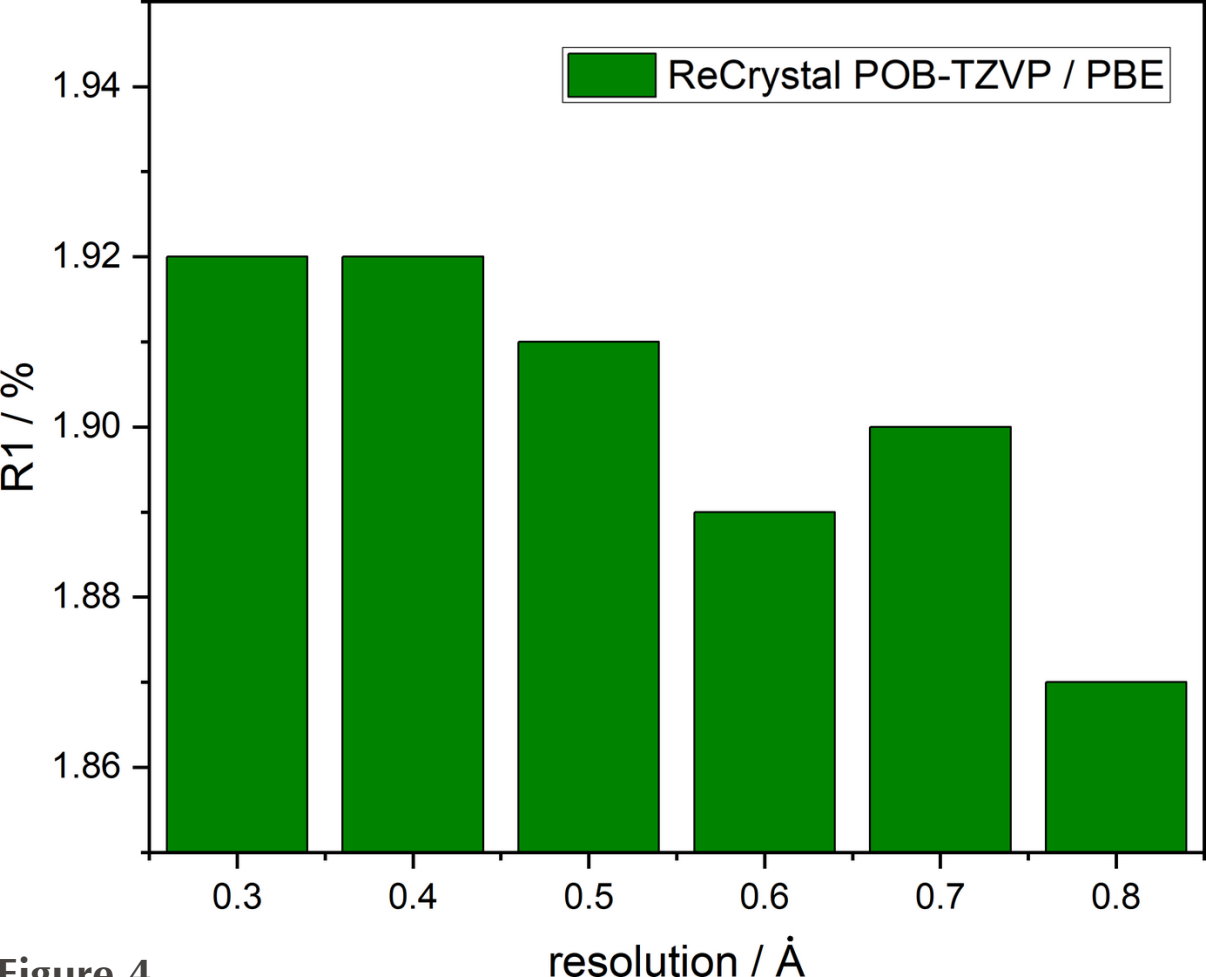
Comparison of the result for the *ReCrystal* refinement at different resolutions for the calculation of theoretical diffraction intensities.

Table 1 ▸ reveals that the result with the lowest *R*1 value is obtained by refinement with the def2-TZVP basis set. *ReCrystal* must be analysed for the behaviour of the refinement and the defined resolution in order to calculate theoretical intensities. The result that fits best is obtained at 0.8 Å (Fig. 4 ▸). This result is explained by the fact that weaker diffraction intensities at resolution values of less than 0.8 Å lead to numerical inaccuracies. In addition, the *ReCrystal* method converges faster at 0.8 Å after seven cycles starting with the IAM geometry and the obtained coordinates are suitable (see xylitol, Section 5).

**Figure 5:**
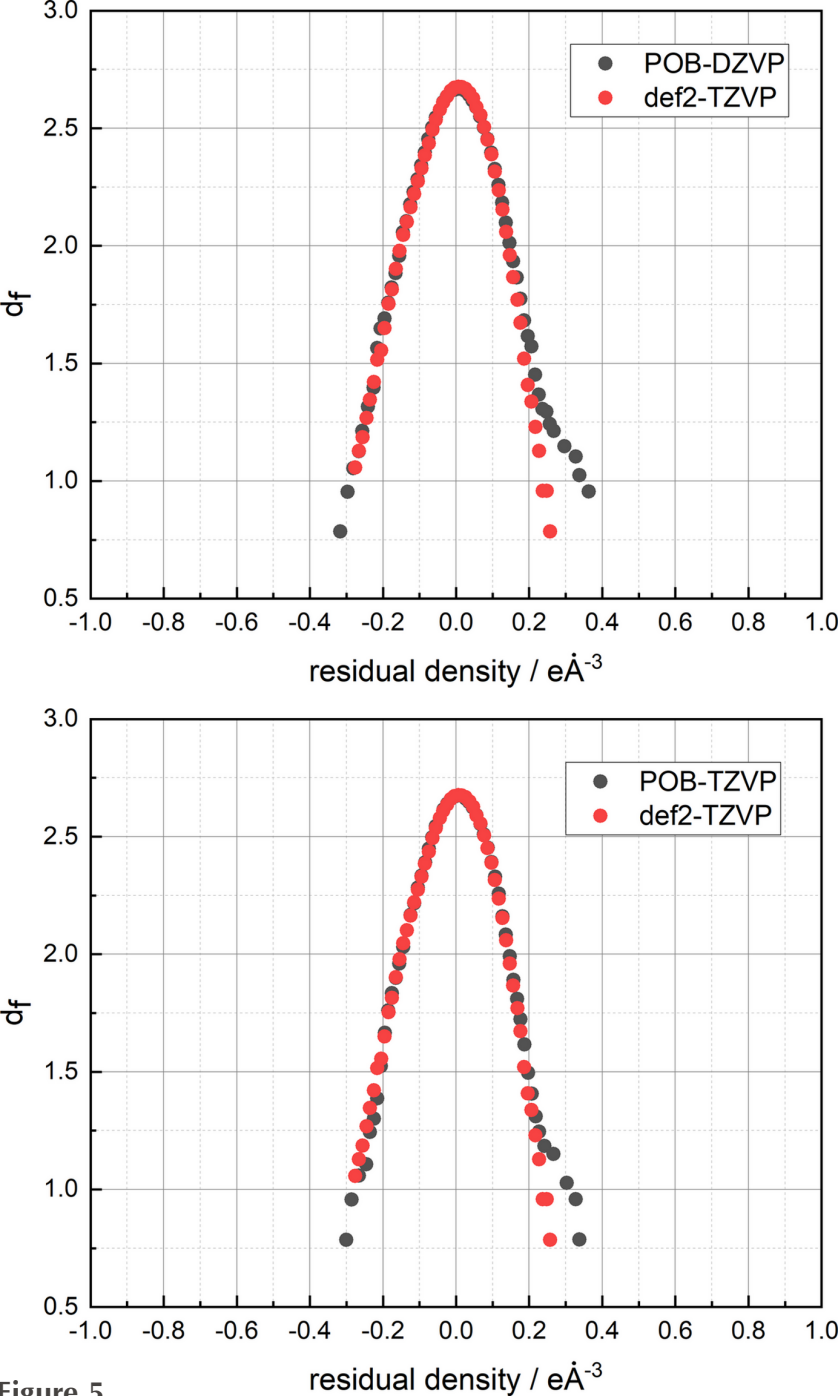
Residual density plot for the *ReCrystal*/0.4 Å refinement of serine for three basis sets: POB-DZVP and POB-TZVP compared with def2-TZVP.

The results of the refinement with different basis sets can be compared using the residual density analysis according to Meindl & Henn (2008 ▸). Fig. 5 ▸ shows that the Ahlrichs basis set def2-TZVP provides a more suitable charge density model than the calculation with POB-TZVP and POB-DZVP.

## 4. Bond distances from *ReCrystal* compared with HAR

The periodic boundary condition used in *ReCrystal* does not lead to any significant difference in the bond distances compared with the refinement using aspherical atomic form factors from a gas phase calculation with HAR. It must be emphasized at this point, however, that *ReCrystal* is based on multipole model atoms and HAR on Hirshfeld atoms. These fundamentally different approaches have significant differences. These include the fact that *ReCrystal* does not use the entire information of the wavefunction and the MMPs cannot exactly reproduce the calculated wavefunction (Bytheway *et al.*, 2002 ▸).

**Figure 6:**
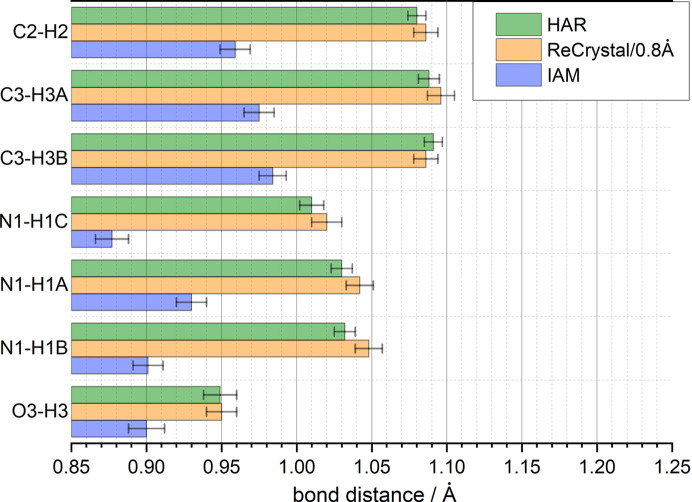
Comparison of bond distances in d/l-serine for the refinement with IAM (*SHELXL*), HAR [NoSpherA2, *ORCA* (version 5.0.3), def2-TZVP/PBE] and *ReCrystal*/0.8 Å (*XD*, *CRYSTAL17*, def2-TZVP/PBE).

Both HAR and *ReCrystal* significantly increase the bond distances to the hydrogen atoms compared with the IAM. This is due to the consideration of the bonding electron density. The bond density is relatively high compared with the total density of the hydrogen atom, so the effect is particularly noticeable for hydrogen. Thus, the hydrogen atom does not have core electrons that can be reasonably described by the IAM (Fig. 6 ▸) (Stewart *et al.*, 1965 ▸) The next step is to compare these results with neutron diffraction. Due to the lack of a neutron diffraction result for d/l-serine, another molecular crystal will be discussed in the next section.

## 5. Comparison to neutron diffraction

**Figure 7:**
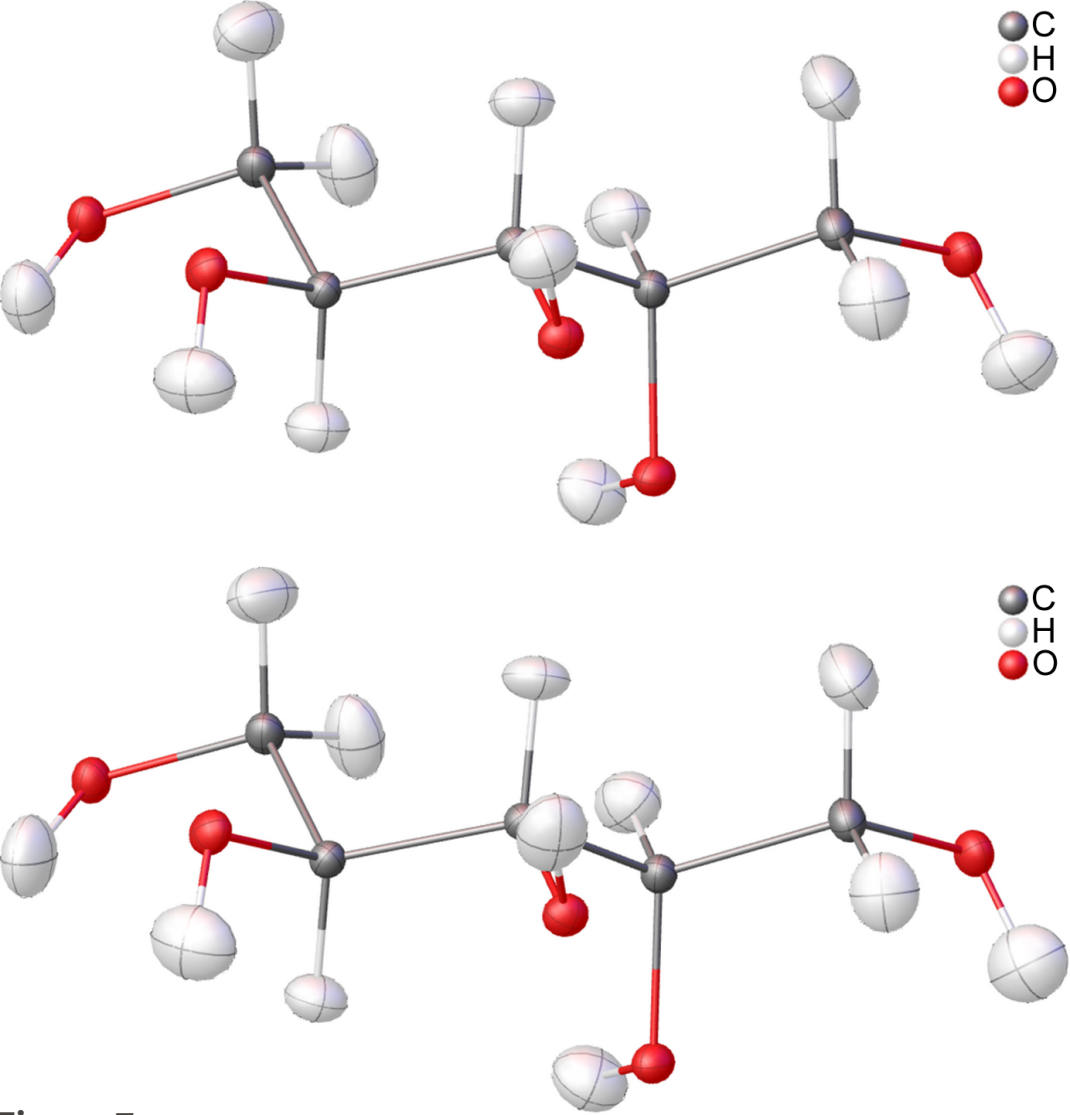
Atomic displacement ellipsoid plot of xylitol for (top) *ReCrystal*/0.8 Å (def2-TZVP/PBE) and (bottom) HAR [NoSpherA2/def2-TZVP/PBE/*ORCA* (version 5.0.3)]; ellipsoid representation at the 50% probability level.

**Figure 8:**
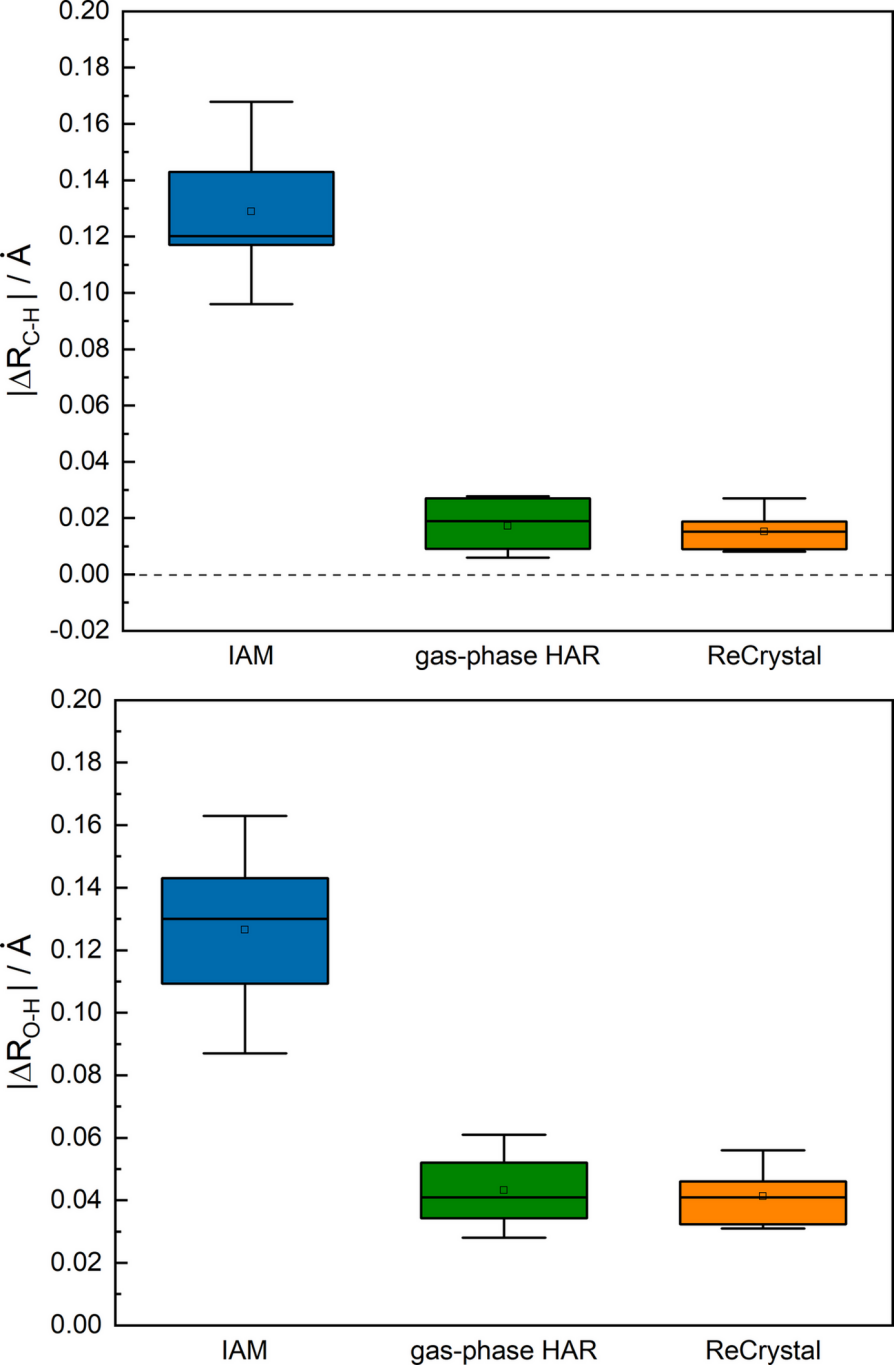
Box-whisker plot analysis showing the difference of bond distances between neutron diffraction and the model for single-crystal X-ray diffraction for xylitol: HAR [NoSpherA2/*ORCA* (version 5.0.3)/def2-TZVP/PBE], *ReCrystal*/0.8 Å (def2-TZVP/PBE) and IAM (*SHELXL*).

The molecular crystal of the achiral organic compound xylitol crystallizes in the Sohncke space group *P*2_1_2_1_2_1_. The crystal structure was first published by Kim & Jeffrey (1969 ▸), who were able to obtain an X-ray diffraction dataset with Cu *K*α radiation. This work is based on a high-resolution dataset (*d*_min_ = 0.41 Å), the dataset for X-ray diffraction with synchrotron radiation at 122 K (CCDC No. 1432562) already published by Madsen *et al.* (2004 ▸). The structure from neutron diffraction at 122 K published by Madsen *et al.* (2003 ▸) (CCDC No. 223330) is also used as a reference (Madsen *et al.*, 2003 ▸). A pronounced network of intermolecular hydrogen bonds is apparent in the crystal structure of the compound. After optimizing the *ReCrystal* procedure, using the basis set def2-TZVP/PBE and calculating tMMP with theoretical intensities up to 0.8 Å, the full performance of *ReCrystal*/0.8 Å is tested if one can obtain reasonable agreement to neutron diffraction bond distances. An extinction correction is used additionally in the iterative refinement, which leads to an improvement in the residual electron density and the *R* values. The extinction correction was applied in each iteration of NoSpherA2/HAR and *ReCrystal*. It was observed that the bond distances are shortened by a few mÅ by introducing the extinction correction (Fig. S6 of the supporting information compared with Fig. 8). Visually, the difference between gas phase HAR and *ReCrystal*/0.8 Å is not obvious from the perspective of the atomic displacement ellipsoid plot (Fig. 7 ▸). Differences can only be found in the shape of the hydrogen ellipsoids bound to oxygen atoms.

**Figure 9:**
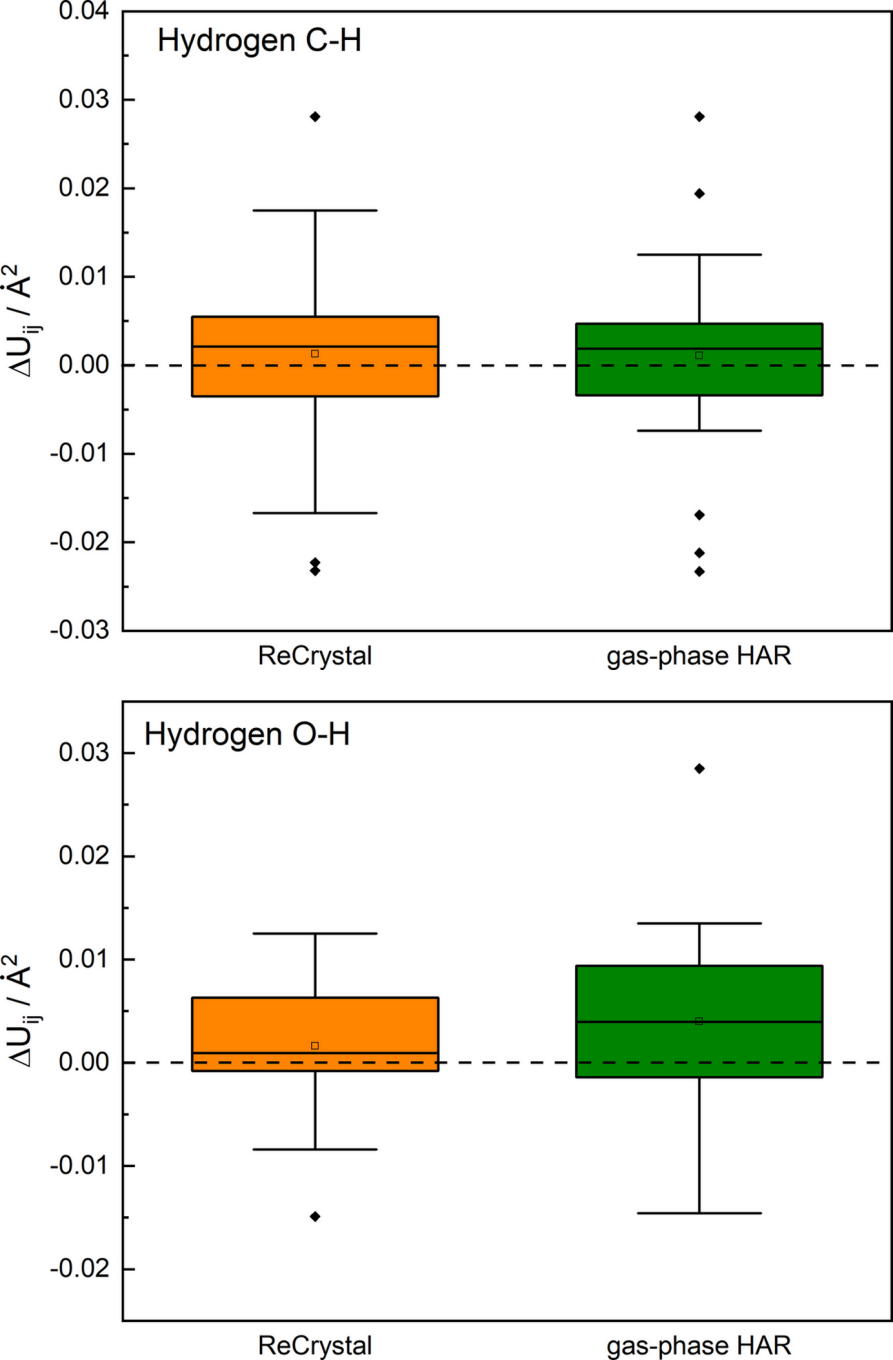
Box-whisker plot analysis showing the difference in the ADPs between neutron diffraction and the model for single-crystal X-ray diffraction for xylitol: HAR [NoSpherA2/*ORCA* (version 5.0.3)/def2-TZVP/PBE] and *ReCrystal*/0.8 Å (def2-TZVP/PBE).

An improvement compared with gas phase HAR (NoSpherA2/def2-TZVP/PBE) can be observed for the determination of the C—H and O—H bond distances (see Fig. 8 ▸). The C—H bond distances can be determined on average with an agreement to neutron diffraction of less than 20 mÅ difference. This example shows that a reasonable determination of the coordinates is possible with *ReCrystal* even though the computational effort is enlarged. In addition, the multipoles can be used for a further refinement of the charge density as shown for serine. Inspection of the fractal dimension analysis of the electron residual density, based on 9790 Bragg intensities, shows that the gas phase HAR is in close agreement with the *ReCrystal* result (Figs. S2 and S3). In addition, it is demonstrated that the anisotropic displacement parameters, especially those of the hydrogen atoms bound to oxygen, are systematically more accurate with *ReCrystal*than with a HAR based on gas phase calculations (Fig. 9 ▸). The interaction density indicates that the polarization is pronounced due to hydrogen bonding on the hydrogen atoms bound to the oxygen (Fig. S5).

## 6. Influence of environmental effects on the molecular charge density

**Figure 10:**
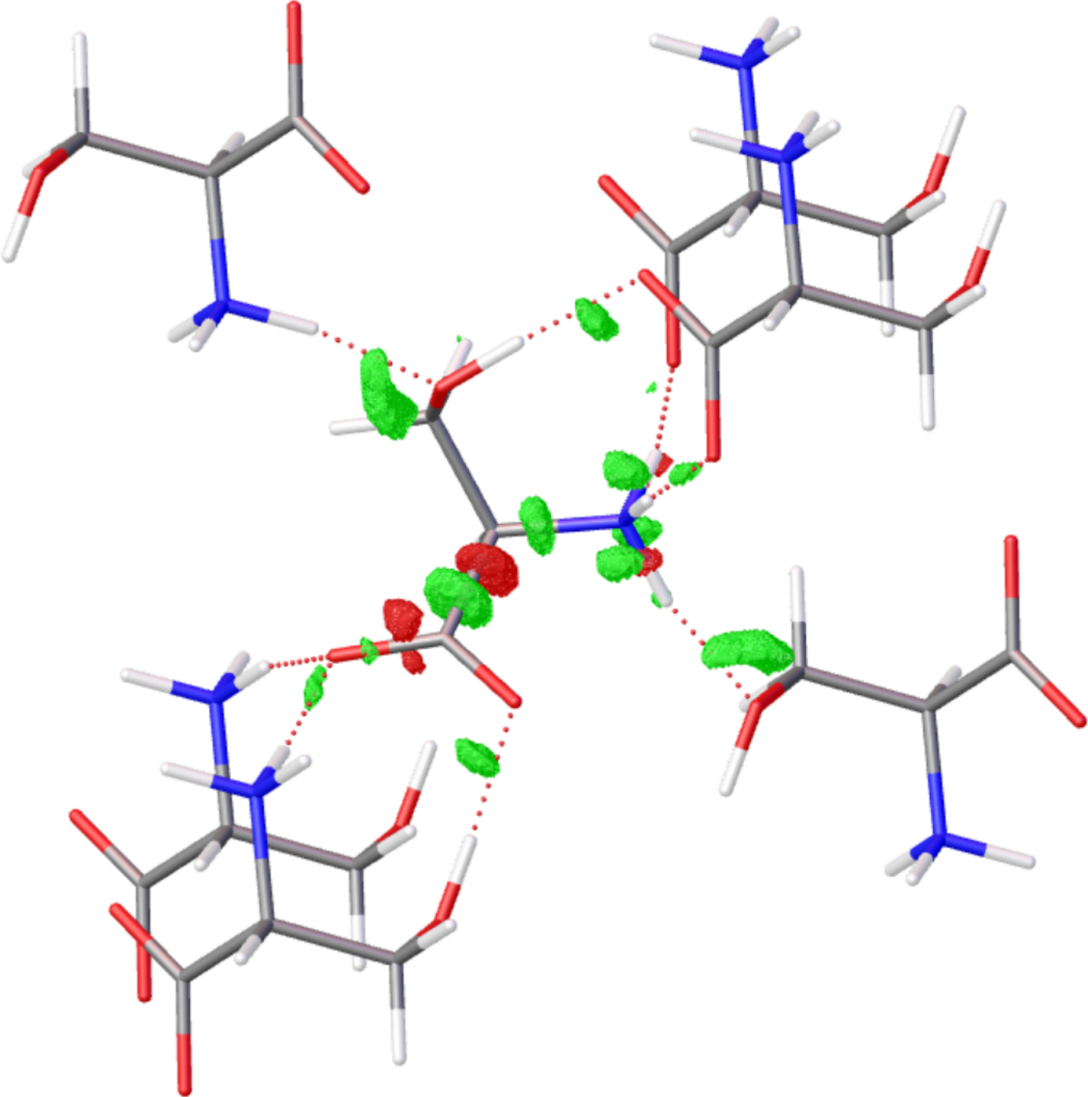
Difference Fourier map for refinement of a synthetic dataset of serine (*CRYSTAL17* def2-TZVP/PBE, *d*_min_ = 0.55 Å) with HAR [isolated molecule, NoSpherA2, *ORCA* (version 5.0), def2-TZVP/PBE] to illustrate the interaction density, isolevel 0.04 e Å^−3^.

From a theoretical point of view, a difference in the charge density of the isolated molecule and the molecule in the crystal can be expected due to the intermolecular interactions (Spackman *et al.*, 1999 ▸). This can be shown in the theoretical difference Fourier map from structure factors generated from isolated molecular densities and interacting molecules (Fig. 10 ▸). The calculated interaction density, which is taken into account when periodic boundary conditions are used in the wavefunction calculation, is illustrated.

The comparison between the *R*1_all_ of the refinement with an isolated charge density from an *ORCA* (version 5.0.3) calculation and the refinement with the periodic density from *CRYSTAL17* shows no significant differences. The *R*1_all_ is 2.28% with HAR and 2.35% with *ReCrystal*/0.4 Å when using the basis set def2-TZVP and the DFT functional PBE. *ReCrystal*/0.8 Å results an *R*1_all_ of 2.23%. The *ORTEP* plots from HAR and *ReCrystal* show only slight differences in the displacement ellipsoids of the hydrogen atoms. Otherwise, no significant structural differences can be observed (Figs. 3 ▸ and 6 ▸).

**Figure 11:**
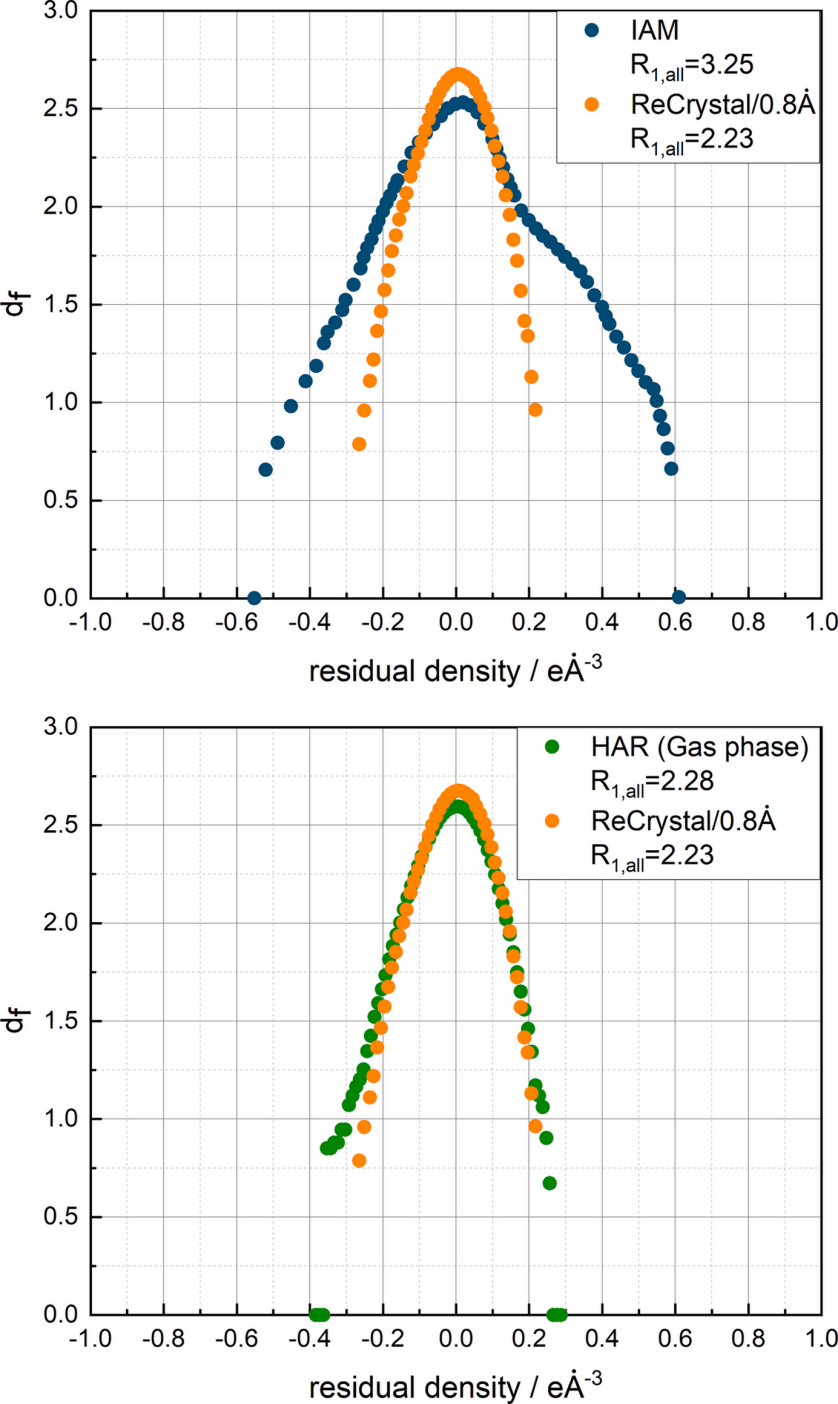
Henn–Meindl residual density plot based on 4930 independent reflections of refinement with *ReCrystal*/0.8 Å, HAR/NoSpherA2 with the basis set def2-TZVP, and functional PBE and IAM for the d/l-serine dataset.

For serine, an improvement in the fractal analysis of the residual density compared with the HAR of the isolated molecule is not evident (see Fig. 11 ▸). The effects of environmental influences on the charge density are not evident. The fractal analysis based on 4930 independent reflections yields the same integrated residual electron density 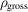 of approximately 11 e Å^−3^. This example is mainly used to test the *ReCrystal* process and to get a general impression of the refinement. It will be necessary to study more systems in order to establish the influence of the charge density by the molecules in the environment that can be observed in an experiment, particularly in the case of extended systems such as coordination polymers and covalent structures.

## 7. Charge density analysis

**Figure 12:**
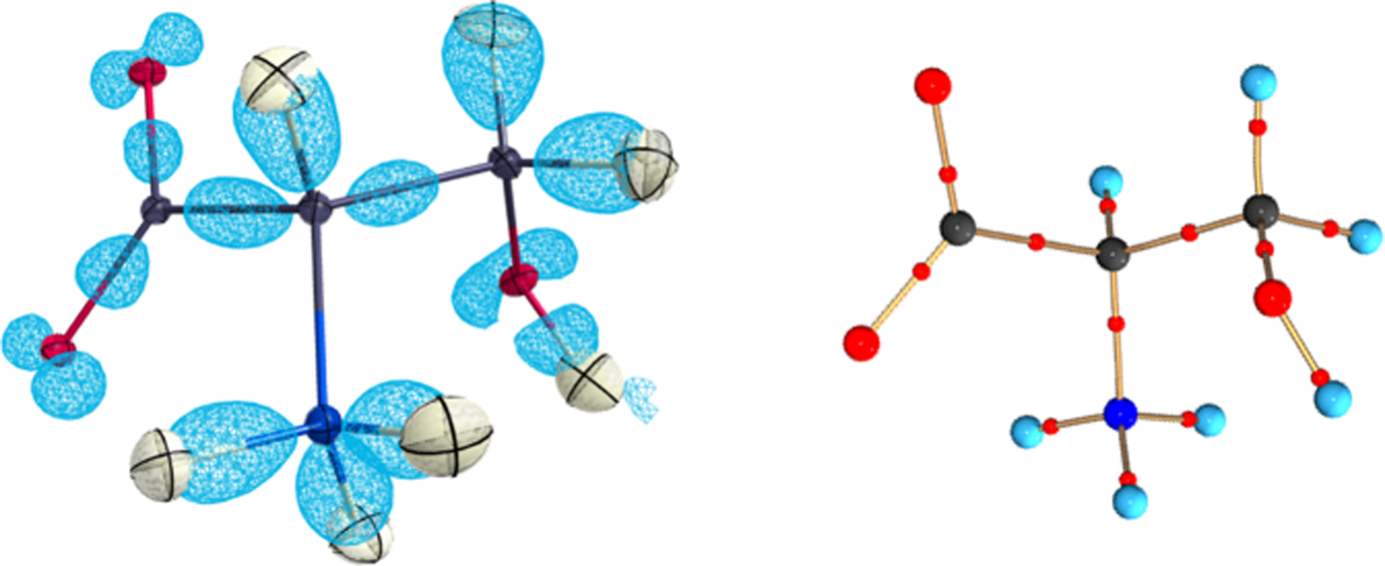
Electron density from the difference between the structure factors of the aspherical model and the spherical model for *ReCrystal* (left: *CRYSTAL17*, def2-TZVP, PBE) and the multipole model based on tMMP from *ReCrystal*/0.8 Å, contour +0.3 e Å^−3^ (ellipsoids represented at the 50% probability level); right: QTAIM analysis for BCPs (red) of the tMMP with *ReCrystal*.

Here we present an advantageous aspect of the Hansen–Coppens model based on *ReCrystal* tMMP in charge density analysis. In particular, *ReCrystal* can be used to analyse weak interactions such as hydrogen bonds. The d/l-serine molecular crystal has been chosen in this case mainly because of the weak molecular interactions in the form of hydrogen bonds in the crystal. Qualitatively, the representation of the deformation density and the representation of the bond critical points (BCPs) can provide an initial impression of the quality of the refinement model (Fig. 12 ▸).

Fig. 10 ▸ shows a positive interaction density in the carbon–nitro­gen bond due to interaction effects. This interaction effect is taken into account in the *ReCrystal* refinement. If the density at the (3,−1) BCP is determined after gas phase HAR for the wavefunction [def2-TZVP/PBE; *Multiwfn* (version 3.6; Lu & Chen, 2012 ▸)], the density is 1.564 e Å^−3^. As expected, the density with *ReCrystal* is higher and is 1.659 e Å^−3^ with the same basis set and functional. The expectation can be additionally checked with *TOPOND17* using the *CRYSTAL17* calculated wavefunction on the POB-TZVP basis level. The value is 1.613 e Å^−3^. This shows that the interaction effects are taken into account in the *ReCrystal* refinement. The interaction density obtained from multipole refinement based on the tMMP of *ReCrystal* converges at 1.626 e Å^−3^.

**Figure 13:**
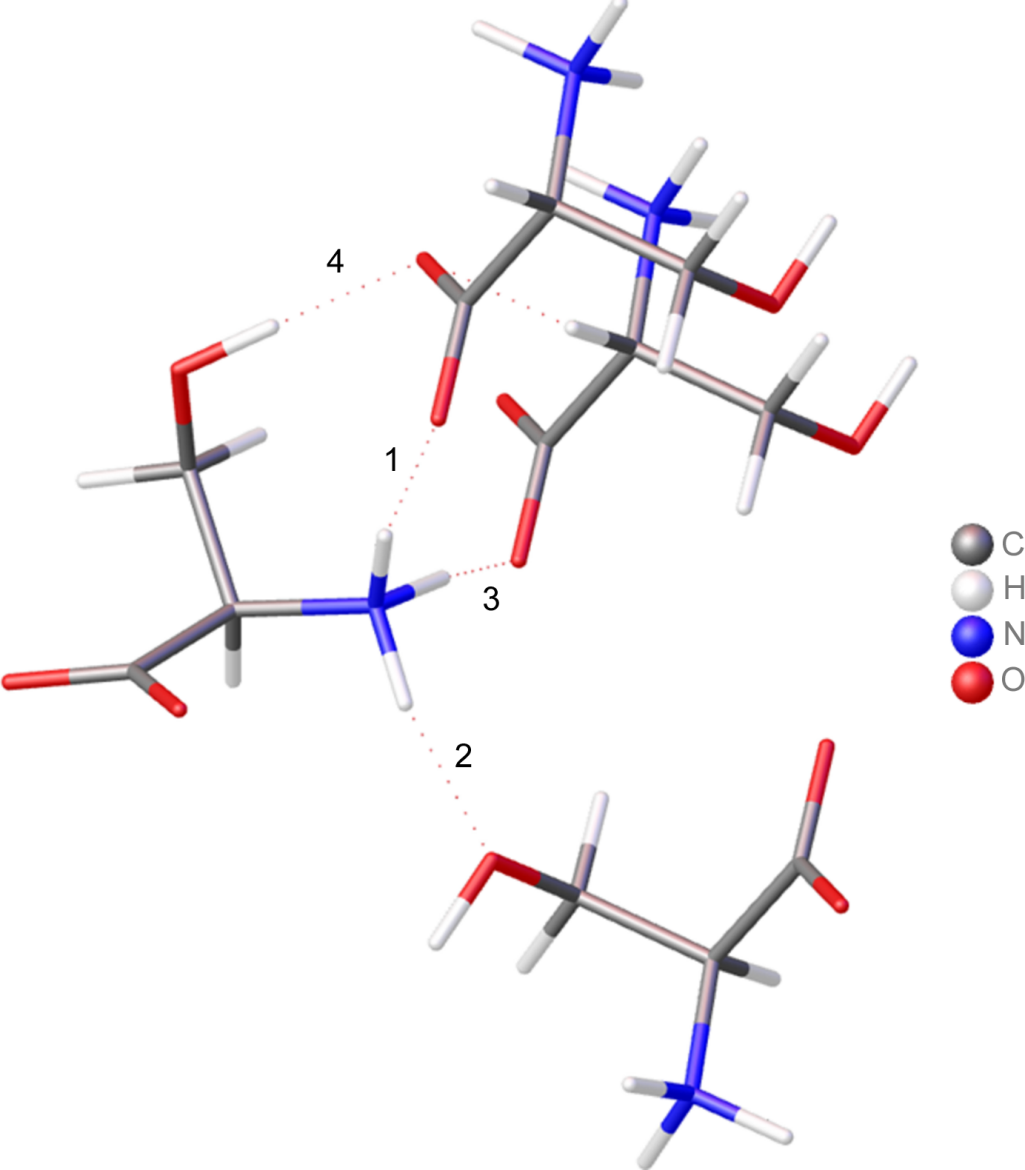
Assignment of hydrogen bonds analysed by QTAIM for verification of the use of the *ReCrystal* tMMP.

The next step is to investigate whether the tMMP of the *ReCrystal* refinement can represent the weak interaction effects of the *CRYSTAL17* wavefunction. To check this, the results of the QTAIM analysis are compared for the weak hydrogen-bonding densities (Fig. 13 ▸ and Table 2 ▸). The *ReCrystal*/0.4 Å tMMP from a calculation def2-TZVP/PBE is analysed with *TOPXD* for the last refinement cycle. The *CRYSTAL17* wavefunction cannot be analysed with *TOPOND17* for the last refinement cycle because *TOPOND17* is not able to analyse *F* functions. This problem is overcome in the latest version of *TOPOND23*, which has not been accessible for us until now. For this reason, the tMMPs from the POB-TZVP/PBE refinement are compared with the *CRYSTAL17* wavefunction. From this point of view one can observe that the values for the charge density can be represented in good agreement with a maximum change of 0.01 e Å^−3^. This value is acceptable with respect to the fact that the interaction density effect occurs in a range slightly higher than 0.01 e Å^−3^. The Laplacian differs slightly compared with the theoretical calculations. This observation has already been described in so far that the multipole model sometimes has problems representing the Laplacian for weak interactions in general (Bytheway *et al.*, 2002 ▸).

In summary, *ReCrystal* refinement with tMMP is a reproduction of the *CRYSTAL17* wavefunction, but with a minor gap of quantitative information. There are several reasons for this situation. One reason is that intensities close to 0 have to be calculated in reciprocal space for the full transformation of the real-space charge density into Bragg intensities, which is generally limited. For this example, *ReCrystal* uses intensities in reciprocal space up to 0.4 Å. Another point is that the MMPs cannot reproduce the charge density exactly because of the use of a tabulated form of exponential coefficients for the radial part. When fitting the theoretical intensity values, *R*1 drops to small values slightly greater than 0%. Nevertheless, the tMMP serves as a reasonable basis for charge density analysis, especially if one is interested in weak interaction effects.

## 8. Outlook and discussion

Multipole model-based iterative refinement (Mbit refinement) is revealed as a new advanced and generally applicable tool in QCr for the refinement of atomic positions and nuclear vibrations. The implementation of iterative refinement with theoretical multipole parameters was presented in the *ReCrystal* procedure (‘Refinement with *CRYSTAL17*’). The resulting coordinates were shown to be comparable to those obtained by HAR. An even better performance than with the gas phase HAR has been observed. Further analysis of the wide range of molecular crystals will be the focus of future research.

Refined multipole parameters in a multipole refinement can account for effects that are not related to bonding electron density [*e.g.* disorder, anharmonic motion (Mallinson *et al.*, 1988 ▸)]. Note that crystals are not ideal objects. Rather, crystals are imperfect systems with dislocations, vacancies and interstitial atoms that affect the diffraction pattern. The major concern is overinterpretation of these crystal construction errors and experimental errors on the observed intensities. Crystal defects can produce undesirable systematic errors in a multipole refinement. From the perspective of experimental charge density investigation, ideally one wants to determine the charge density from the diffraction pattern in order to investigate the true bonding situation. At this point, theory can contribute decisively to the study of systems of interest. Theoretical multipole parameters, which can be generated in the *ReCrystal* code, generate parameters that are already optimized and avoid the risk of describing defects caused by non-ideal properties of a crystal in a charge density study using the conventional multipole model. *ReCrystal* thus represents a modern approach to the study of charge density, combining the best of both experimental and theoretical techniques.

However, there are limitations to overcome. One problem arises when refining atoms on special positions. Because of the use of *XD2006*, special care must be taken when processing atoms on special positions. This problem no longer exists in *XD2024*, so this program will have to be incorporated into *ReCrystal* in future. In addition, refinement with *ReCrystal* and *CRYSTAL17* is more computationally intensive than simple HAR. This is mainly due to the fact that after calculating the periodic wavefunction, a synthetic dataset must be created from which the aspherical atom form factors are then generated.

**Figure 14:**
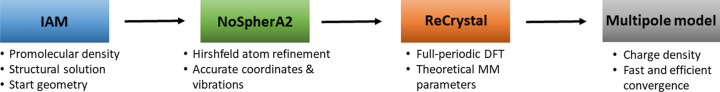
Optional flowchart for *ReCrystal* refinement of molecular crystals; *ReCrystal* also provides access to experimental charge density investigations on a theoretical basis with optimized coordinates and displacement parameters.

The combination of the *ReCrystal* refinement with a preceding HAR has proven to be particularly advantageous (Fig. 14 ▸). Starting from the HAR structure, the *ReCrystal* refinement converges faster to the minimum and reliably produces a charge density model that can reduce the residual charge density compared with IAM. It should also be possible to combine the *ReCrystal* refinement with the normal mode refinement developed by Hoser & Madsen (2016 ▸).

So far, this method has been tested intensively on molecular systems (Patzer, 2023 ▸). Note that the *ReCrystal* code has since been the subject of optimization and testing. This method can be used in particular for systems whose binding motifs are periodic in nature such as hydrogen-bonding motifs, coordination polymers and covalent structures. This is the first step for future research to explore the full periodic charge density in the crystalline solid state with the newly developed method based on periodic boundary calculations. It has already been established from a theoretical perspective that the *ReCrystal* process also works for these periodic systems to refine hydrogen atom positions, particularly those involved in intermolecular hydrogen bonds. This has been clearly demonstrated by comparisons with the results of neutron diffraction.

## 9. Related literature

The following reference is cited in the supporting information: Larsen (1985 ▸).

## Supplementary Material

CIFs and related data for the refinements of serine and xylitol. DOI: 10.1107/S2052252525002040/fc5082sup1.zip

Supporting figures and output files. DOI: 10.1107/S2052252525002040/fc5082sup2.pdf

CCDC references: 2431127, 2431128, 2431129, 2431130

## Figures and Tables

**Table 1:** Dependence of *R*1/% and *wR*2/% in the *ReCrystal*/0.4 Å refinement on the choice of the basis set and DFT functional; refinement of D/L-serine (rac.) ten cycles A dash refers to convergence not fulfilled in *CRYSTAL17*.

	PBE		BLYP		B3LYP		CAM-B3LYP		wB97		M062X	
	*R*1	*wR*2	*R*1	*wR*2	*R*1	*wR*2	*R*1	*wR*2	*R*1	*wR*2	*R*1	*wR*2
POB-DZVP	1.99	4.61	1.98	4.59	1.96	4.56	2.04	4.76	–	–	1.97	4.58
6-31G	1.93	4.45	1.92	4.43	1.92	4.42	1.92	4.42	1.93	4.43	1.92	4.42
def2-SVP	1.93	4.47	1.92	4.44	1.92	4.43	1.92	4.42	1.92	4.43	1.93	4.45
POB-DZVPP	1.92	4.46	1.91	4.43	–	–	–	–	–	–	–	–
POB-TZVP	1.91	4.45	1.91	4.44	1.90	4.41	1.89	4.39	1.89	4.41	1.90	4.41
def2-TZVP	1.87	4.36	1.87	4.36	–	–	–	–	–	–	–	–

**Table 2:** BCPs in the crystal structure of D/L-serine for intermolecular bonds First row: free multipole refinement (MM, *XD2016*, *VM database*, *TOPXD*); second and third row: *ReCrystal*/0.4 Å (PBE, *TOPXD*); fourth row: *TOPOND* from the *CRYSTAL17* wavefunction (POB: POB-TZVP/PBE; def2: def2-TZVP/PBE).

	Bond	*D* (Å)	*D*_1_ (Å)	*D*_2_ (Å)	CP	ρ_CP_ (e Å^−3^)	∇^2^ρ_CP_ (e Å^−5^)	λ_1_ (e Å^−5^)	λ_2_ (e Å^−5^)	λ_3_ (e Å^−5^)	ε
1	MM	1.823	0.638	1.185	(3,−1)	0.228	2.142	−1.530	−1.255	4.928	0.219
	*ReCrystal* def2	1.819	0.649	1.170	(3,−1)	0.252	2.040	−1.533	−1.447	5.020	0.060
	*ReCrystal* POB	1.814	0.639	1.175	(3,−1)	0.248	2.207	−1.513	−1.388	5.108	0.090
	*TOPOND* POB				(3,−1)	0.249	2.482	−1.398	−1.350	5.229	0.026
2	MM	1.793	0.635	1.158	(3,−1)	0.264	2.766	−1.377	−1.250	5.393	0.101
	*ReCrystal* def2	1.794	0.648	1.146	(3,−1)	0.267	2.710	−1.474	−1.398	5.582	0.054
	*ReCrystal* POB	1.793	0.641	1.152	(3,−1)	0.265	2.745	−1.458	−1.364	5.567	0.069
	*TOPOND* POB				(3,−1)	0.250	2.940	−1.446	−1.350	5.736	0.071
3	MM	1.805	0.638	1.167	(3,−1)	0.248	2.174	−1.653	−1.263	5.090	0.309
	*ReCrystal* def2	1.798	0.643	1.155	(3,−1)	0.256	2.405	−1.556	−1.416	5.377	0.099
	*ReCrystal* POB	1.823	0.653	1.170	(3,−1)	0.239	2.465	−1.381	−1.253	5.100	0.102
	*TOPOND* POB				(3,−1)	0.229	2.771	−1.253	−1.205	5.229	0.037
4	MM	1.731	0.596	1.135	(3,−1)	0.335	1.439	−2.329	−2.191	5.959	0.063
	*ReCrystal* def2	1.726	0.599	1.127	(3,−1)	0.323	1.767	−2.121	−2.079	5.967	0.021
	*ReCrystal* POB	1.720	0.586	1.134	(3,−1)	0.302	2.204	−1.996	−1.916	6.115	0.042
	*TOPOND* POB				(3,−1)	0.290	3.181	−1.687	−1.663	6.531	0.024
