# Supplementary material for: Solid-state calculations for iterative refinement in quantum crystallography using the multipole model
Source: IUCrJ. 2025 Apr 4;12(Pt 3):322–33. doi: 10.1107/S2052252525002040 (PMC12044847; doi:10.1107/S2052252525002040)
Supplement: Supplementary file 2 [file m-12-00322-sup2.pdf]

IUCrJ

Volume 12 (2025)

Supporting information for article:

Solid-state calculations for iterative refinement in quantum crystallography using the multipole model

Michael Patzer and Christian W. Lehmann

Solid-state calculations for iterative refinement in quantum crystallography using the multipole model

Michael Patzer and Christian W. Lehmann

Max-Planck-Institut für Kohlenforschung, Kaiser-Wilhelm-Platz 1, Mülheim an der Ruhr, 45470, Germany.

patzer@kofo.mpg.de

Supporting Information

Flow Chart for ReCrystal

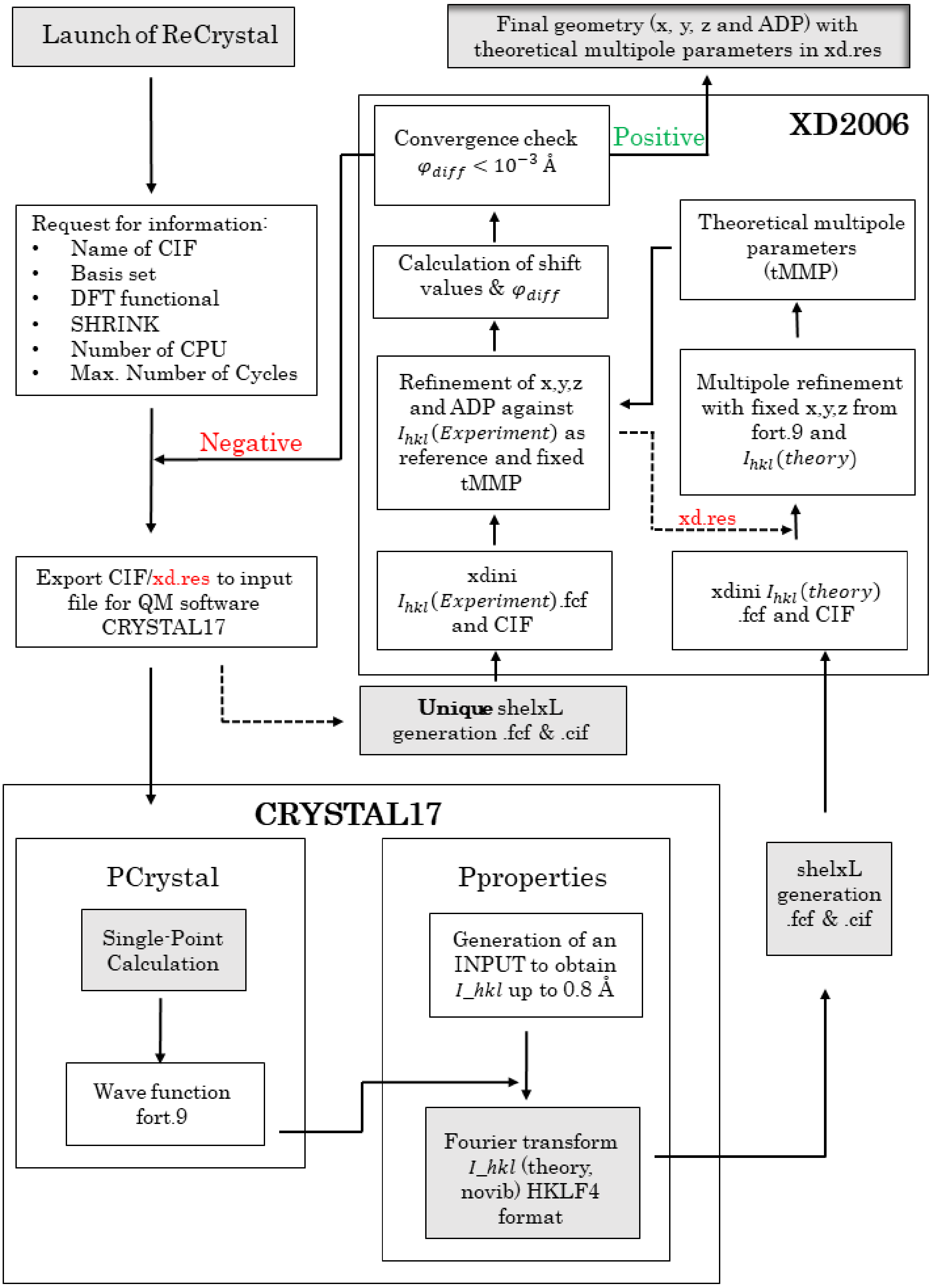

Figure S1 Detailed sequence of a ReCrystal/0.8Å refinement, ReCrystal acts as a coordinator between the programs ShelxL, XD2006 and CRYSTAL17.

Refinement of Xylitol

ReCrystal/0.8Å: 6 cycles are required for refinement until the convergence criterion is met.
The output files of ReCrystal are shown below:

ReCryst_convergence.log:

This file contains the convergence parameter that is calculated by ReCrystal after each cycle.

```
Refinement started at:  2025-02-11 19:07:35.327082

Cycle        RMS-shift:  xyz(Angstroem)
--------------------------------------------------
  2                      0.009163454410395958
  3                      0.0012637395763930707
  4                      0.0002767041711776329
  5                      0.00011184232924778717
  6                      6.64193881631002e-05
--------------------------------------------------
Refinement finished at: 2025-02-11 20:45:02.615097
```

ReCryst.out

```
###########################  INPUT PARAMETERS
##################################

   BASIS SET       : def2-TZVP
   DFT FUNCTIONAL  : PBE
   SHRINK          : 8 8
   Number of Procs : 48
   MAX Num. Cycles : 10
############################## DOKU R-Values
###################################

Refinement started at:  2025-02-11 19:07:35.327082
----------------------------------------------------------------------------
----
Residuals after final cycle

R{ F } =     0.0147                                Rw{ F } =     0.0136
R{F^2} =     0.0185                                Rw{F^2} =     0.0270
GOFw  =     0.6872          GOF =     0.6872     Nref/Nv =    41.3500
----------------------------------------------------------------------------
----
Residuals after final cycle

R{ F } =     0.0147                                Rw{ F } =     0.0137
R{F^2} =     0.0176                                Rw{F^2} =     0.0272
GOFw  =     0.6922          GOF =     0.6922     Nref/Nv =    41.3500
----------------------------------------------------------------------------
----
Residuals after final cycle

R{ F } =     0.0142                                Rw{ F } =     0.0131
R{F^2} =     0.0164                                Rw{F^2} =     0.0260
GOFw  =     0.6611          GOF =     0.6611     Nref/Nv =    41.3500
----------------------------------------------------------------------------
----
Residuals after final cycle
```

```
R{ F } =     0.0142                                  Rw{ F } =     0.0131
R{F^2} =     0.0166                                  Rw{F^2} =     0.0260
GOFw  =     0.6625           GOF =     0.6625     Nref/Nv =    41.3500
------------------------------------------------------------------------
----
Residuals after final cycle

R{ F } =     0.0142                                  Rw{ F } =     0.0131
R{F^2} =     0.0166                                  Rw{F^2} =     0.0260
GOFw  =     0.6620           GOF =     0.6620     Nref/Nv =    41.3500
------------------------------------------------------------------------
----
Residuals after final cycle

R{ F } =     0.0142                                  Rw{ F } =     0.0131
R{F^2} =     0.0166                                  Rw{F^2} =     0.0260
GOFw  =     0.6618           GOF =     0.6618     Nref/Nv =    41.3500
------------------------------------------------------------------------
----

Refinement finished at :2025-02-11 20:45:02.615097
```

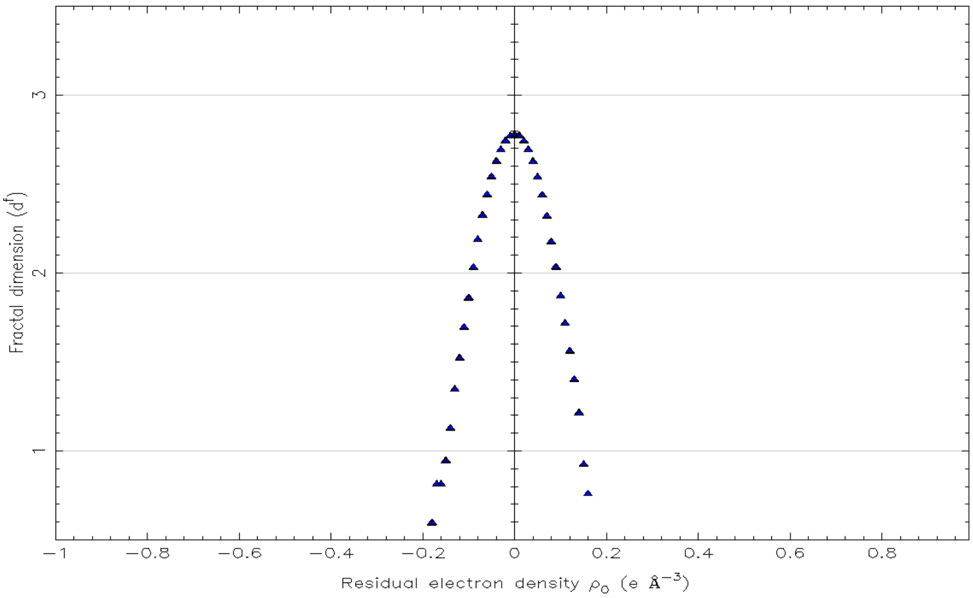

Figure S2 Henn-Meindl-Plot for the refinement of Xylitol with ReCrystal/0.8Å (def2-TZVP/PBE) based on 9790 reflexes.

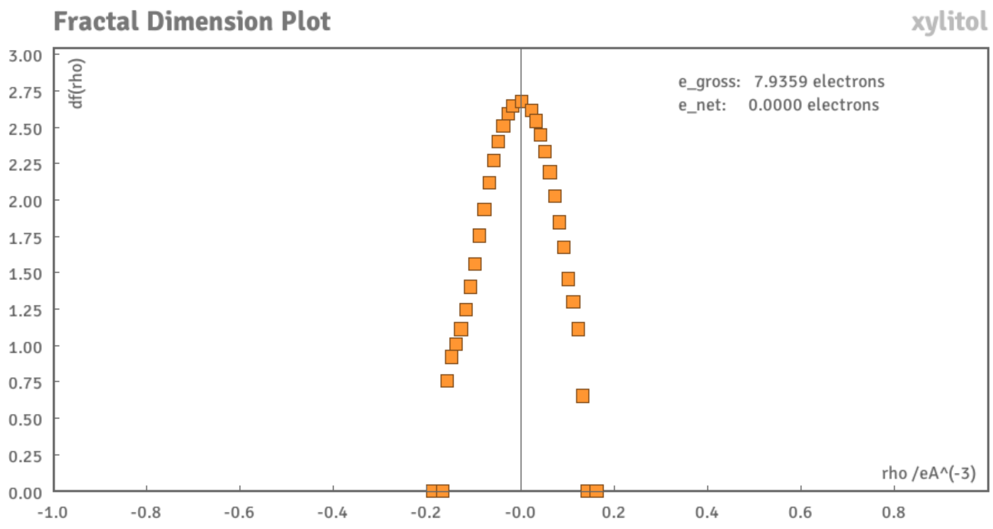

Figure S3 Henn-Meindl-Plot for the refinement of Xylitol with NoSpherA2 (def2-TZVP/PBE/ORCA 5.0.3) 9790 reflexes.

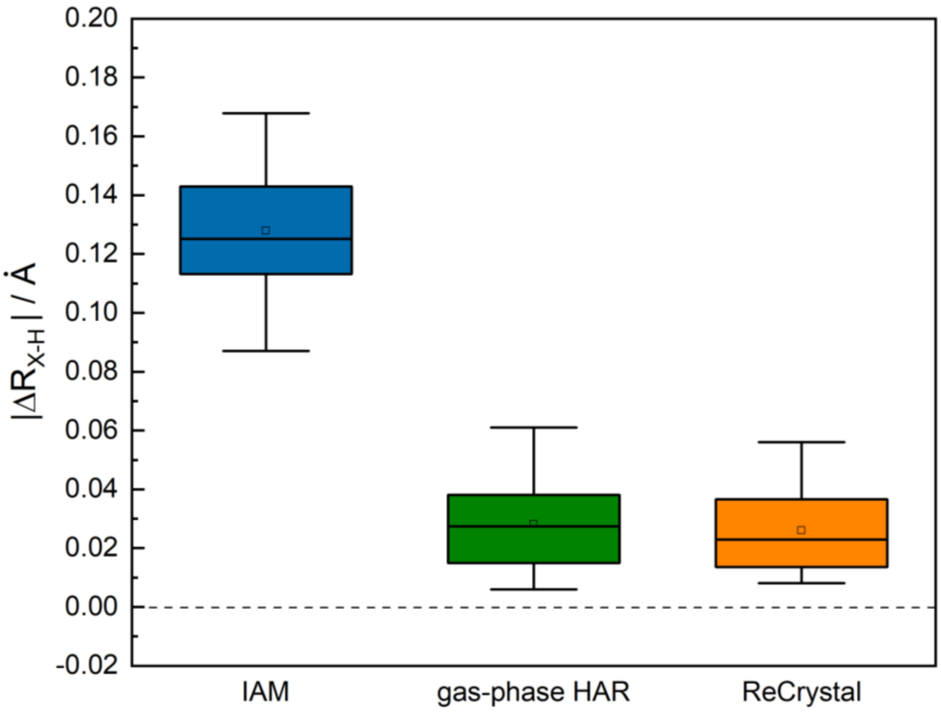

Figure S4 Box-Whisker-Plot for the refinement of Xylitol with IAM (shelxL), gas-phase HAR (NoSpherA2, def2-TZVP,PBE) and ReCrystal/0.8Å (def2-TZVP/PBE) with reference to the neutron diffraction experiment mentioned in the manuscript (X: Oxygen and Carbon).

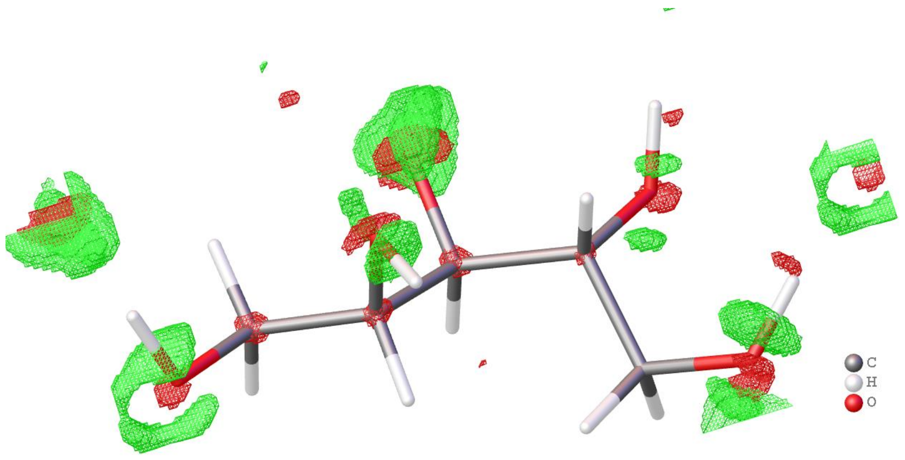

Figure S5 Difference Fourier Map for refinement of a synthetic data set of Xylitol (CRYSTAL17 def2-TZVP/PBE, dmin = 0.60 Å) with HAR (isolated molecule, NoSpherA2, ORCA5.0, def2-TZVP/PBE) to illustrate the interaction density, isolevel 0.03 e/Å^3, ellipsoids representation on 50 % probability level.

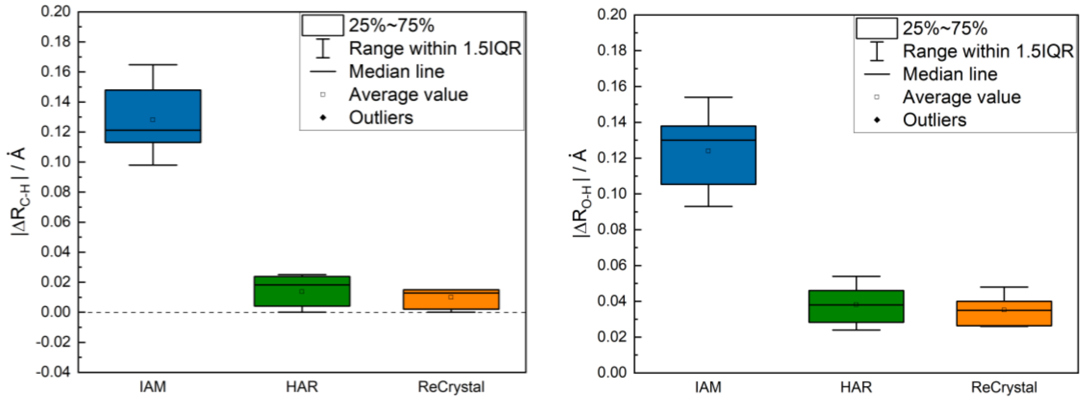

Figure S6 Xylitol: Box-Whisker-Plot analysis showing the difference of bond distances between neutron diffraction and the model for single crystal X-ray diffraction for xylitol HAR (NoSpherA2/ORCA 5.0.3 / def2-TZVP/PBE); ReCrystal/0.8Å (def2-TZVP/PBE) and IAM (shelxL) **without extinction correction**.

Rejection of reflections - sigobs criterium

The XD refinement takes into account the "sigobs" criterion that selects the weak reflections (default "sigobs 3.0 1.E06"). This is an automatic part of the XDINI routine to set the value of sigobs. In the direct comparison with NoSpherA2 and the IAM, for example the residual electron density (Henn-Meindl plot), sigobs is set to the value "0.0 1.E06" (in XDLSM and XDFFT). It is important to note that this rejection only affects the R value, not the structural model (xyz and ADP). This was verified and can be explained with the weighting scheme (see master file of XD, xd.mas), which selects the weak reflections in the refinement already, regardless of the sigobs criterion. For this reason, sigobs was set to "0.0 1.E06" only in the comparison of R-values and residual electron densities. This ensures that the results are consistent when compared.

Refinement of D/L-Serine

ReCrystal/0.8Å: 8 cycles are required for refinement until the convergence criterion is met.
The output files of ReCrystal are shown below:

ReCryst_convergence.log:

```
Refinement started at:  2025-02-12 15:15:29.868933

Cycle        RMS-shift:  xyz(Angstroem)
--------------------------------------------------
  2                      0.018960960465667677
  3                      0.0039052765306200827
  4                      0.0021707550118399608
  5                      0.0022938846985417452
  6                      0.0004252648094578394
  7                      0.00013521015388751195
  8                      7.970782103847816e-05
--------------------------------------------------
Refinement finished at: 2025-02-12 16:20:31.063507
```

ReCryst.out

```
##########################  INPUT PARAMETERS
##################################

   BASIS SET       : def2-TZVP
   DFT FUNCTIONAL  : PBE
   SHRINK          : 8 8
   Number of Procs : 32
   MAX Num. Cycles : 10
############################### DOKU R-Values
###################################
Refinement starts at : 2025-02-12 15:15:29.868933
-------------------------------------------------------------------------
-------
Residuals after final cycle

R{ F } =     0.0241                              Rw{ F } =     0.0274
R{F^2} =     0.0430                              Rw{F^2} =     0.0553
GOFw  =     1.6956          GOF =     1.6956     Nref/Nv =    32.1181
-------------------------------------------------------------------------
-------
Residuals after final cycle

R{ F } =     0.0180                              Rw{ F } =     0.0213
R{F^2} =     0.0330                              Rw{F^2} =     0.0433
GOFw  =     1.3262          GOF =     1.3262     Nref/Nv =    32.1181
-------------------------------------------------------------------------
-------
Residuals after final cycle

R{ F } =     0.0177                              Rw{ F } =     0.0208
R{F^2} =     0.0324                              Rw{F^2} =     0.0423
GOFw  =     1.2984          GOF =     1.2984     Nref/Nv =    32.1181
```

```
--------------------------------------------------------------------------
-------
Residuals after final cycle

R{ F } =     0.0178                              Rw{ F } =     0.0210
R{F^2} =     0.0324                              Rw{F^2} =     0.0426
GOFw  =     1.3070          GOF =     1.3070     Nref/Nv =    32.1181
--------------------------------------------------------------------------
-------
Residuals after final cycle

R{ F } =     0.0176                              Rw{ F } =     0.0207
R{F^2} =     0.0319                              Rw{F^2} =     0.0420
GOFw  =     1.2883          GOF =     1.2883     Nref/Nv =    32.1181
--------------------------------------------------------------------------
-------
Residuals after final cycle

R{ F } =     0.0175                              Rw{ F } =     0.0207
R{F^2} =     0.0319                              Rw{F^2} =     0.0420
GOFw  =     1.2888          GOF =     1.2888     Nref/Nv =    32.1181
--------------------------------------------------------------------------
-------
Residuals after final cycle

R{ F } =     0.0175                              Rw{ F } =     0.0207
R{F^2} =     0.0319                              Rw{F^2} =     0.0420
GOFw  =     1.2883          GOF =     1.2883     Nref/Nv =    32.1181
--------------------------------------------------------------------------
-------
Residuals after final cycle

R{ F } =     0.0175                              Rw{ F } =     0.0207
R{F^2} =     0.0319                              Rw{F^2} =     0.0420
GOFw  =     1.2881          GOF =     1.2881     Nref/Nv =    32.1181
--------------------------------------------------------------------------

Refinement finished at :2025-02-12 16:20:31.063507
```

Box-Whisker-Plot

The box-whisker plot (also box-and-whisker plot) is a visualisation method for data value distributions.[1] The plot is used in this work to analyse the deviation of the structural models from the refinement to the neutron diffraction experiment. The following values are included in the visualisation:

Percentile
The percentile X (X assumes values from 0.01 to 0.99, 1 % to 99 %) can be assigned a specific data value of the data set. Below this value are X*100 % of all data in the data set.

IQR - Interquartile range
The IQR is defined as the difference between the data value at X=0.75 and X=0.25.

Median
The median is the value at 50 % of all data.

Mean value
Arithmetic mean of the values of all data points

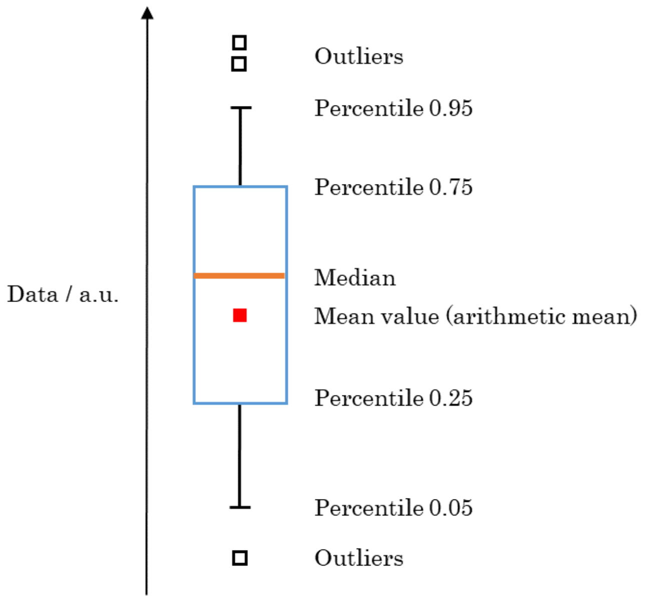

Figure S7 Qualitative box-whisker plot as generated from a data set in the Origin program [2]; in the illustration, the range between the percentile 0.25 and 0.75 is outlined with a box.

1. Larsen, R. D., Box-and-whisker plots. Journal of Chemical Education 1985, 62 (4), 302.
2. OriginLab 17.2.2 Creating Box Charts. https://www.originlab.com/doc/Origin-Help/Create-Box-Chart (accessed 17.01.2025, 08:51 UTC).
